# Long Short-term Memory–Based Prediction of the Spread of Influenza-Like Illness Leveraging Surveillance, Weather, and Twitter Data: Model Development and Validation

**DOI:** 10.2196/42519

**Published:** 2023-02-06

**Authors:** Maria Athanasiou, Georgios Fragkozidis, Konstantia Zarkogianni, Konstantina S Nikita

**Affiliations:** 1 School of Electrical and Computer Engineering National Technical University of Athens Zografos, Athens Greece

**Keywords:** influenza-like illness, epidemiological surveillance, machine learning, deep learning, social media, Twitter, meteorological parameters

## Abstract

**Background:**

The potential to harness the plurality of available data in real time along with advanced data analytics for the accurate prediction of influenza-like illness (ILI) outbreaks has gained significant scientific interest. Different methodologies based on the use of machine learning techniques and traditional and alternative data sources, such as ILI surveillance reports, weather reports, search engine queries, and social media, have been explored with the ultimate goal of being used in the development of electronic surveillance systems that could complement existing monitoring resources.

**Objective:**

The scope of this study was to investigate for the first time the combined use of ILI surveillance data, weather data, and Twitter data along with deep learning techniques toward the development of prediction models able to nowcast and forecast weekly ILI cases. By assessing the predictive power of both traditional and alternative data sources on the use case of ILI, this study aimed to provide a novel approach for corroborating evidence and enhancing accuracy and reliability in the surveillance of infectious diseases.

**Methods:**

The model’s input space consisted of information related to weekly ILI surveillance, web-based social (eg, Twitter) behavior, and weather conditions. For the design and development of the model, relevant data corresponding to the period of 2010 to 2019 and focusing on the Greek population and weather were collected. Long short-term memory (LSTM) neural networks were leveraged to efficiently handle the sequential and nonlinear nature of the multitude of collected data. The 3 data categories were first used separately for training 3 LSTM-based primary models. Subsequently, different transfer learning (TL) approaches were explored with the aim of creating various feature spaces combining the features extracted from the corresponding primary models’ LSTM layers for the latter to feed a dense layer.

**Results:**

The primary model that learned from weather data yielded better forecast accuracy (root mean square error [RMSE]=0.144; Pearson correlation coefficient [PCC]=0.801) than the model trained with ILI historical data (RMSE=0.159; PCC=0.794). The best performance was achieved by the TL-based model leveraging the combination of the 3 data categories (RMSE=0.128; PCC=0.822).

**Conclusions:**

The superiority of the TL-based model, which considers Twitter data, weather data, and ILI surveillance data, reflects the potential of alternative public sources to enhance accurate and reliable prediction of ILI spread. Despite its focus on the use case of Greece, the proposed approach can be generalized to other locations, populations, and social media platforms to support the surveillance of infectious diseases with the ultimate goal of reinforcing preparedness for future epidemics.

## Introduction

### Background

Infectious diseases are ranked among the top 10 leading causes of death worldwide, affecting particularly young children in low-income countries [1], and impose an undue burden on a socioeconomic and political level that is felt worldwide. Rapidly changing environments, increased globalization, evolving socioeconomic conditions, and varying lifestyles accompanied by the genetic flexibility of different infectious agents influence the epidemic potential of infectious diseases and render them a long-standing, emerging, and reemerging global health threat [2]. Seasonal influenza (also referred to as *flu*) is a common infectious disease caused by influenza viruses that appears as the rapid onset of a combination of systemic and respiratory symptoms [3]. It is generally characterized by a fast human-to-human transmission rate and presents with pandemic, epidemic, or seasonal patterns, with epidemic outbreaks occurring every year. It is recognized as a leading cause of acute respiratory illnesses, increasing morbidity and mortality and resulting in approximately 3 to 5 million cases of severe illness and 290,000 to 650,000 respiratory deaths annually, whereas a much higher mortality rate is usually reported during pandemic periods [4].

The impact of influenza and influenza-like illness (ILI) epidemics is highly dependent on the overall vulnerability of the population, with most healthy individuals usually presenting with mild symptoms and a quick recovery [4]. High-risk groups, including individuals with chronic conditions such as cardiovascular disease, hypertension, asthma, diabetes, and rheumatoid arthritis as well as older adults, are more likely to experience severe or lethal infections. The presence of comorbidities and complications can influence the severity of the disease, thus significantly affecting hospitalization and death rates. It is estimated that a higher demand for health care services and increased health care costs during an influenza pandemic can impose an economic burden of up to US $60 billion every year [3]. Moreover, increased levels of work and school absenteeism because of influenza and ILI outbreaks account for financial and productivity losses, thus challenging economies and societies worldwide [5].

Effective public health surveillance is recognized as the cornerstone of public health decision-making for the early detection and prevention of epidemics caused by infectious diseases such as seasonal influenza. It envisages the monitoring of known infectious diseases based on their epidemic potential, timely identification of emerging infections, and monitoring and early management of antimicrobial drug resistance. International coordination and collaboration along with an enhanced response at the national level are required for the establishment of a timely, complete, and sensitive public health surveillance policy [6]. However, several limitations have fragmented public health surveillance systems, thus hampering the adoption of an integrated and well-coordinated strategy for the prevention and control of infectious diseases [7]. These are mainly related to inefficient data management, inadequate computing resources, poor adoption of advanced analytic tools, workforce shortages, and financial barriers. Novel approaches for addressing such multifaceted challenges leverage advances in IT, data analytics, and sharing, which, combined with the expansion of available data sources, create high expectations and encourage the exploration of new frontiers in public health surveillance [8,9].

Within this framework, the concepts of *infodemiology* and *infoveillance* have emerged, describing the use of internet data along with traditional data sources (eg, epidemiological data) for the development of digital disease detection (DDD) systems [2]. DDD systems harness the power of artificial intelligence coupled with the exploitation of heterogeneous data sources, including electronic health records, internet search engine queries, social media queries, crowdsourced event-based information queries, and geolocation and weather data, to monitor and predict the spread of infectious diseases [10]. In this way, performance limitations owing to delays in the collection of timely and accurate data, which is a problem frequently encountered by conventional surveillance approaches, can be eliminated. It is expected that the integration of DDD into conventional public health surveillance systems will strengthen public health prevention with the ultimate goal of building healthier communities.

The recent COVID-19 pandemic has spurred a paradigm shift in the adoption of artificial intelligence in disease surveillance, uncovering the potential of DDD to enhance safety, efficiency, and effectiveness and, ultimately, contribute to the successful control of the pandemic [11]. Along these lines, a number of research endeavors have been directed toward the development of epidemiological models for predicting the spread of COVID-19 infections. These have been fueled by machine learning and deep learning techniques and traditional as well as alternative data sources, which enable the timely detection of disease footprints [11-14].

### Related Work

Considering their high pandemic potential, ILIs have attracted wide interest as a case study for the development of DDD systems. Several computational models have been proposed for forecasting ILI cases leveraging various combinations of data sources and data analytics methods. The potential to increase the predictive performance of these models through the consideration of alternative data sources along with epidemiological data [15] has triggered a substantial number of relevant research studies in which the use of search engine queries [16], Wikipedia access logs [17], social media data [18] such as Twitter and Facebook data [19], weather data [20,21], and geolocation data [22] has been explored. Moreover, the combination of multiple alternative data sources and their integration with traditional (ie, epidemiological) data has been investigated as a means of further enhancing the predictive power of these models [15].

Along these lines, the first research attempt dates back to 2006, when anonymous search engine queries were used for the estimation of ILI incidence [23]. The Google Flu Trends system, which followed in 2008, proved to be a landmark in DDD. It leveraged users’ search patterns to calculate ILI infection rates but failed to provide accurate estimations despite evidence of its ability to supplement conventional surveillance approaches [24]. Since then, a multitude of methodologies in the areas of statistics, machine learning, deep learning, and graph mining have been deployed along with a variety of alternative data toward the development of computational models able to detect, nowcast, or forecast ILI outbreaks [25,26]. Literature has documented different approaches, investigating the use of logistic regression, random forests, decision trees, k-nearest neighbor regression, artificial neural networks, support vector machine (SVM) regression, Bayesian networks, long short-term memory (LSTM) recurrent neural networks (RNNs), and convolutional neural networks [27-30]. Among these, logistic regression, SVM, and artificial neural networks have gained the most widespread acceptance because of their simplicity and good predictive ability.

It is noteworthy that the use of Twitter data has gained increased popularity with respect to the various alternative data sources and has been shown to substantially improve the predictive performance of ILI models over similar baseline models that only consider epidemiological data such as historical ILI data [31]. Many studies have analyzed Twitter data in different languages, such as Japanese, Arabic, and English, to support ILI surveillance [26,30,32-34]. State-of-the art natural language processing (NLP) techniques have been combined in these studies with various machine learning approaches, including decision trees, SVM, and LSTM, for predicting ILI spread. Thus, Twitter opens up new opportunities for the development of data-richer models, enabling the timely monitoring of ILI trends and the accurate prediction of ILI outbreaks. The ease of downloading, archiving, and preprocessing Twitter’s ever-flowing textual data through its application programming interface (API) constitutes an additional attractive element that has led many researchers to explore the potential of ILI-related tweets (ie, tweets including self-reports about the influenza status and recovery process). More recently, several research endeavors have used Twitter data for the implementation of digital approaches aiding COVID-19 surveillance. Emphasis has been placed on the application of various NLP techniques for topic modeling and sentiment analysis, whereas only a few studies have used Twitter data to predict the evolution of COVID-19 cases [34-37]. It is worth noting that little previous research has focused on the use case of the Greek language within the context of modeling disease spread from Twitter data, whereas NLP resources and methods are less well developed for Greek than for English [38].

The effect of weather conditions on ILI spread has also attracted considerable interest, and substantial studies have proposed that certain meteorological parameters such as temperature, humidity, rainfall, UV radiation, sunshine duration, and wind speed have an impact on ILI seasonal patterns and spread [39,40]. ILI prediction models leveraging either Twitter or weather data have exhibited varying performances, whereas the combined use of Twitter and weather data for the development of a computational model able to accurately predict ILI cases has not been investigated.

In this work, a deep learning approach based on LSTM was deployed for the development of prediction models able to nowcast (estimation of the current week) and forecast official weekly ILI cases. Data on the population and weather conditions of Greece were used for the development and evaluation of the models. The potential of alternative data sources to enhance ILI surveillance was investigated through the consideration of different combinations of heterogeneous data, including ILI surveillance data, Twitter data, and a set of meteorological parameters. To the best of the authors’ knowledge, this is the first work harnessing the combined power of Twitter and weather data along with deep learning for the accurate prediction of ILI cases.

## Methods

### Ethical Considerations

No ethics approval was required for this study as it was not considered to be human participant research. The ILI epidemiological data were obtained from publicly available reports. Developer accounts were granted by Twitter to the authors of this study, enabling access to Twitter data. The granted API keys were used in a way that was compliant with the Twitter Developer Agreement and Policy. Direct and indirect identifier data fields and information such as usernames, comments, number of likes, photos, or videos were excluded upon data collection.

### Data Sets

#### Overview

The development and evaluation of the proposed ILI prediction models were based on the combined use of multiple data sources. In particular, weekly ILI surveillance data, Twitter data, and weather data for Greece corresponding to the period from 2010 to 2019 were collected and considered to compose the models’ input space. Considering that the study’s focus was strongly placed on investigating the potential of social data to enhance ILI prediction and given that, after 2020, the outbreak of COVID-19 dominated public interest reflected in social media discussions, data referring to the decade before the outbreak of COVID-19 were used to better serve the study objectives.

#### ILI Surveillance Data

The clinical surveillance of ILI by the European Influenza Surveillance Network is generally based on weekly reports devised by sentinel general practitioners [41]. Most sentinel surveillance systems report the number of new cases of ILI or acute respiratory infection. Data from laboratory- and hospital-based surveillance are considered in these weekly surveillance updates. In particular, specimens coming from sentinel sites as well as sources of nonsentinel surveillance (eg, hospitals, nonsentinel physicians, and clinics) are analyzed by national reference laboratories that report influenza test results. Moreover, information about laboratory-confirmed influenza-positive hospitalized cases is considered. Geographic distribution and adequate representation in terms of age, sex, ethnicity, socioeconomic status, and risk factors (eg, existence of chronic disease) are considered in national sentinel site selection. Thus, data made available through sentinel surveillance provide a reliable estimate of the actual ILI spread.

**Figure 1:**
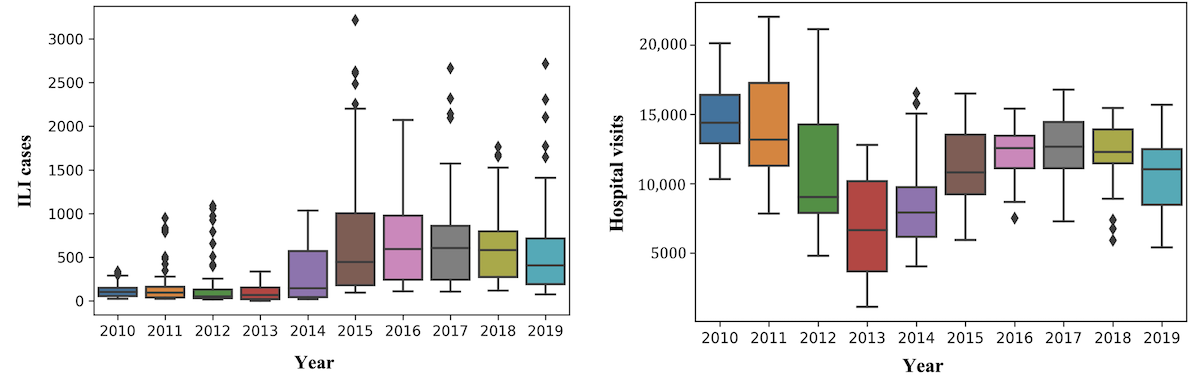
Distribution of influenza-like illness (ILI) cases (left panel) and hospital visits (right panel) per year throughout the decade of 2010 to 2019.

Within the framework of this study, ILI-related epidemiological data, including the number of ILI-related hospital visits and the number of ILI cases reported in Greece on a weekly basis between January 2010 and December 2019 (520 weeks in total), were collected from the publicly available reports of the Hellenic National Public Health Organization, which is responsible for the surveillance and control of infectious diseases in Greece [42]. Figure 1 illustrates the distribution of the weekly number of ILI cases and hospital visits throughout each year of the period from 2010 to 2019.

#### Weather Data

**Figure 2:**
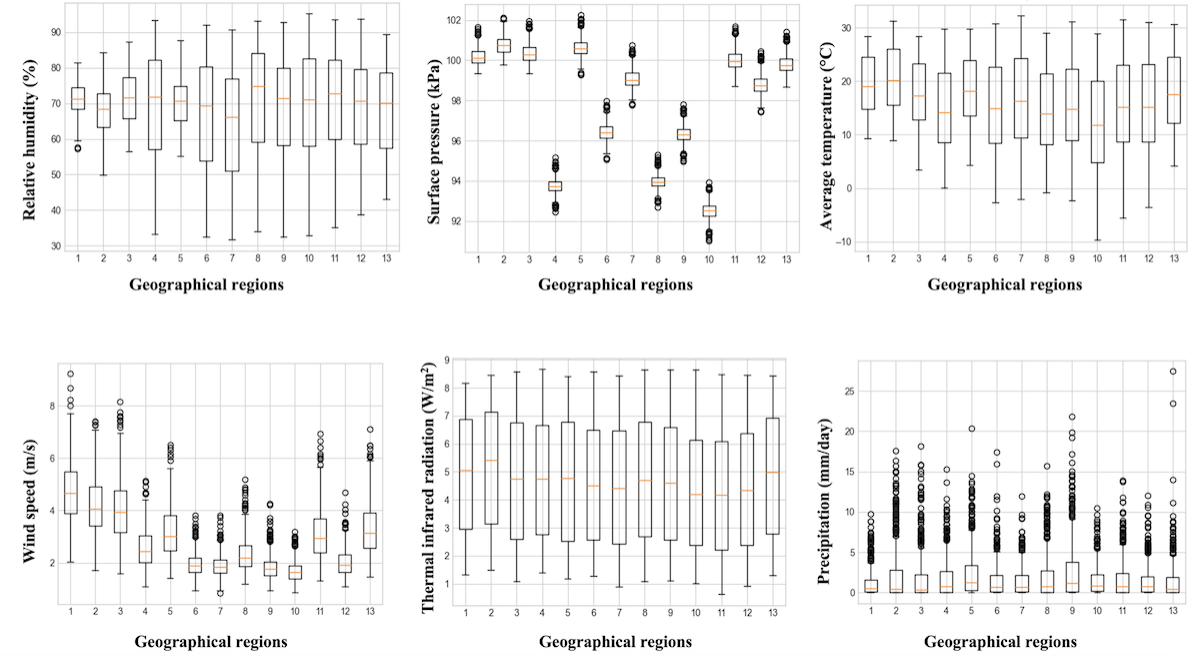
Distribution of the considered weekly meteorological parameters throughout the decade of 2010 to 2019 for the 13 Greek geographical regions. 1: East Macedonia and Thrace; 2: Central Macedonia; 3: Western Macedonia; 4: Epirus; 5: Thessaly; 6: Ionian Islands; 7: Western Greece; 8: Central Greece; 9: Attica; 10: Peloponnese; 11: North Aegean; 12: South Aegean; 13: Crete.

Considering the influential nature of weather conditions on the evolution of ILI spread, different meteorological parameters were considered to enrich the models’ input space. To this end, the National Aeronautics and Space Administration web-based application, which provides access to real-time solar and meteorological data, was used for the generation of weekly meteorological reports corresponding to the period of 2010 to 2019 [43]. The obtained data set included weekly measurements of relative humidity; surface pressure; minimum, maximum, and average temperature; average wind speed; thermal infrared radiation; and precipitation across the 13 geographical regions of Greece. Figure 2 shows the distribution of these measurements for each geographical region throughout the decade of 2010 to 2019.

#### Twitter Data

##### Collection

Twitter data were collected by deploying the Python library *GetOldTweets3* [44]. The publicly available Twitter filtered streaming API was not selected to be used because of its data acquisition rate limit. Given that most Twitter users in Greece post tweets in Greek language, only tweets in Greek were selected to be considered for composing the Twitter data set. The filtering of tweets was based on geographic location (ie, all geographic regions of Greece), period (ranging between January 2010 and December 2019), and a set of ILI-related keywords in Greek that were defined with the aim of collecting posts that featured ILI-related content such as users’ self-reports about their ILI infection and recovery status and reflections on ILI spread within the community. The applied set of keywords, translated into English, is presented in Textbox 1. All tweets containing at least one of the predefined keywords were collected. Subsequently, the Python library *Tweepy* was deployed for filtering out tweets outside Greece [45]. Using the unique ID of each tweet post, fetched by GetOldTweets, an object model was retrieved for each tweet, and the value of the location attribute was used for identifying tweets from Greece. In case the value of the corresponding attribute was null, an instance of the user object related to the tweet post of interest was obtained, and the user’s location was considered as the tweet’s location.

The final collected set included 337,923 tweets, which were anonymized in terms of usernames, user identification numbers, and tweet identification numbers to ensure user privacy. Tweets potentially associated with social bots were excluded from the data set through the deployment of the Botometer API [46].

**Figure 3:**
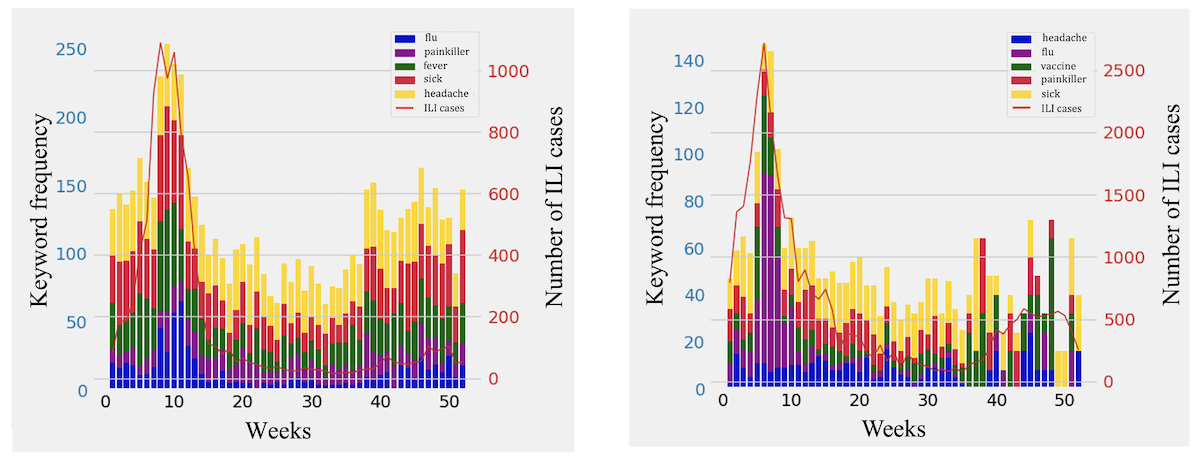
The progression of the frequency of appearance of the most predominant influenza-like illness (ILI)–related keywords in the collected tweets per week for the years 2012 and 2019. The set of the most frequently encountered ILI-related keywords was differentiated throughout the decade.

A statistical analysis based on the use of the Pearson correlation coefficient (PCC) was performed to explore potential correlations between the progression of the number of ILI-related keywords in the Twitter data set and the number of ILI cases per week. Statistically significant correlations (PCC >0.5 and *P*<.05) were identified in all the years of the decade under study except for the years 2010, 2011, and 2014, thus unveiling the existence of reflections of the ILI epidemic curve in Twitter use. The progression of the frequency of appearance of the most dominant ILI-related keywords in the collected weekly tweet set with respect to weekly ILI cases for the years 2012 and 2019 is presented in Figure 3.

**Keywords used for the collection of Twitter posts related to influenza-like illness.**

- Disease-related keywords
  flu
  H1N1
  virus
  gastroenteritis
  otitis
  asthma
  cold
- Symptom-related keywords
  cough
  pain
  diarrhea
  dizziness
  migraine
  nausea
  respiratory
  sneeze
  stomachache
  symptoms
  fever
  sick
  ill
  sore throat
  headache
  chill
  shiver
- Treatment-related keywords
  antibiotics
  aspirin
  paracetamol
  ibuprofen
  medicine
  painkillers
  painkiller brands
  pill
  recover
  vaccine

##### Preprocessing

Appropriate preprocessing of the obtained Twitter data set was carried out before feature extraction. In particular, the exact geographical location of each of the remaining tweets was identified in terms of the 13 Greek regions using the GeoPy (Python Software Foundation) client [47]. Moreover, the NLP open-source Python library *spaCy* along with the Greek language pipeline *el_core_news_md* were used for tokenization; conversion to lowercase characters; and removal of punctuation, URLs, hashtags, numbers, emojis, noninformative words (eg, articles and pronouns), and tokens. The latter were defined as words that were not included in the predefined set of keywords and appeared 5 times in the corpus of the collected tweets. Stop words were also removed to eliminate low-level information from the collected data, and a lemmatization process was followed to replace inflected words with their common dictionary form. All words were then converted to their corresponding word embeddings, each represented by a 1×300 array, using the word2vec model, which is implemented in the spaCy library [48]. Moreover, bigram features, defined as a set of 2-word sequences appearing in the corpus of the obtained tweets, were extracted.

##### Feature Extraction Based on the Semantic Clustering of Words and Bigrams

Considering the character limit in Twitter posts in combination with the circumlocutory nature and semantic complexity of the Greek language, emphasis was placed on the investigation of different preprocessing techniques for the extraction of highly informative features. First, standard preprocessing techniques such as stemming and lemmatization were applied for extracting frequently used text features, including word n-grams (unigrams, bigrams, and trigrams), term frequency–inverse document frequency scores, and text embeddings. However, their use led to low predictive performance, thus highlighting the need to apply a more sophisticated preprocessing method. In this direction, word clusters were created with the aim of mapping semantic correlations between words and identifying highly informative ILI-related terms across the obtained Twitter data set. Moreover, semantic clustering facilitated the computational efficiency of the training procedure by contributing to dimensionality reduction in the case of Twitter’s input space. In particular*,* spectral clustering was applied to the generated word embeddings [49]. According to this approach, the set of word embeddings was transformed into a similarity graph based on the calculation of the cosine similarity between the word embeddings, 
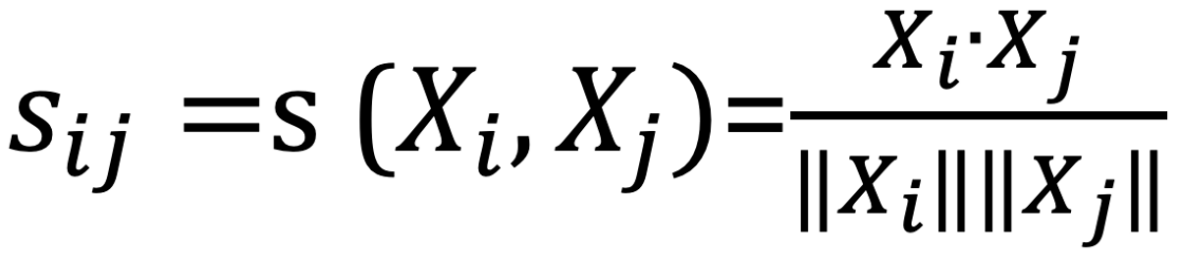
 (1), where *s_ij_* is the cosine similarity between the word embeddings *X_i_* and *X_j_*.

The graph nodes were subsequently mapped to a low-dimensional space (ie, a partition of the graph characterized by weights with lower values on the edges between different groups and higher values on the edges within a group). To this end, the Laplacian matrix *L* was constructed as follows: *L* = *D* – *W* (2), where *D* is the degree matrix, which is a diagonal matrix featuring information regarding the number of edges attached to each node, and *W* is the weighted adjacency matrix, which encapsulates the edge weight information. The first 30 eigenvectors *v_k_* (*k*=1...30) of the normalized *L* were then calculated and used as columns for building matrix *U*, which was normalized according to the following relationship: 
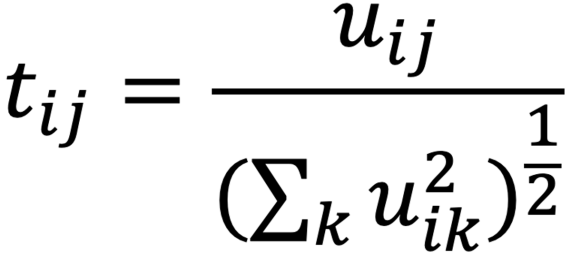
 (3), where *u_ij_* is the element of the *i*th row and *j*th column of *U*; *k*=1, 2,..., 30; and *t_ij_* is the element of the *i*th row and *j*th column of the resulting matrix *T.* The rows of matrix *T* were subsequently grouped into 30 clusters based on the *k*-means algorithm. The elbow method was deployed to select the optimal number of word clusters. The aforementioned process led to the formation of 30 clusters of word embeddings.

Special emphasis was placed on the clustering of ILI-related keywords, which conveyed significant information regarding the users’ health status. In particular, the predefined ILI-related keywords, which were used for the retrieval of Twitter data, were transformed into their corresponding cluster assignments ranging from 1 to 30. Considering that data collection was performed on a weekly basis, the frequency of appearance of the ILI-related keyword embeddings across the weekly Twitter data set was subsequently estimated.

The resulting clusters of word embeddings were used to convert the bigrams into embedding vectors. In particular, the words included in each bigram were replaced with their corresponding cluster assignments, thus being converted into 2D vectors with elements spanning the value range [1, 30]. In this way, 124 unique vectors representing unique combinations of the 2 clusters were identified. Subsequently, similar to the procedure followed for the ILI-related keywords, the frequency of appearance of the 124 bigram embeddings across each weekly Twitter data set was calculated.

##### Additional Features

Apart from the calculated frequencies of appearance of the ILI-related keyword embeddings and bigram embeddings, other features were also used to compose the input space of the computational models for nowcasting and forecasting the weekly ILI cases. In particular, considering that the collection of Twitter data was performed on a weekly basis across the 13 geographic regions of Greece, the weekly number of unique Twitter users and the weekly number of tweets in the obtained data set were also calculated for each geographic region.

##### Seasonal Decomposition of the Extracted Features

As the extracted weekly features from the collected Twitter data were characterized by complex seasonal patterns, reflecting the variance in the number of ILI cases throughout the year, a seasonal decomposition strategy was adopted with the aim of minimizing the complexity of the extracted time-series features, thus facilitating the computational models’ learning process. In particular, the seasonal trend decomposition (STD) method was applied, enabling the identification of seasonal components along with nonperiodic changes in the extracted features [50]. The former refers to the repeating patterns existing in the time-series data, which are associated with the yearly evolution of ILI cases and may change slowly over time, whereas the latter accounts for the introduced randomness, mainly because of changes in the yearly Twitter use rate in Greece. According to STD, a time series is decomposed into the *trend*, *seasonality*, and *residual* components based on the following relationship: 
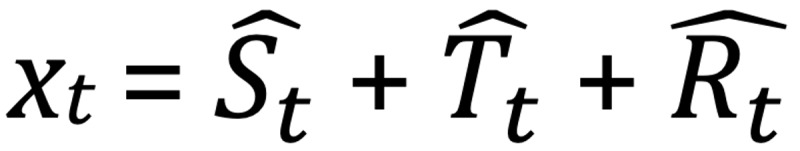
 (4), where *x_t_* is an observation at time *t* and 
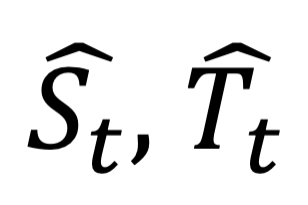
 and 
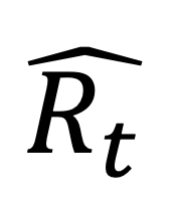
 refer to the *seasonality*, *trend*, and *residual* components, respectively.

**Figure 4:**
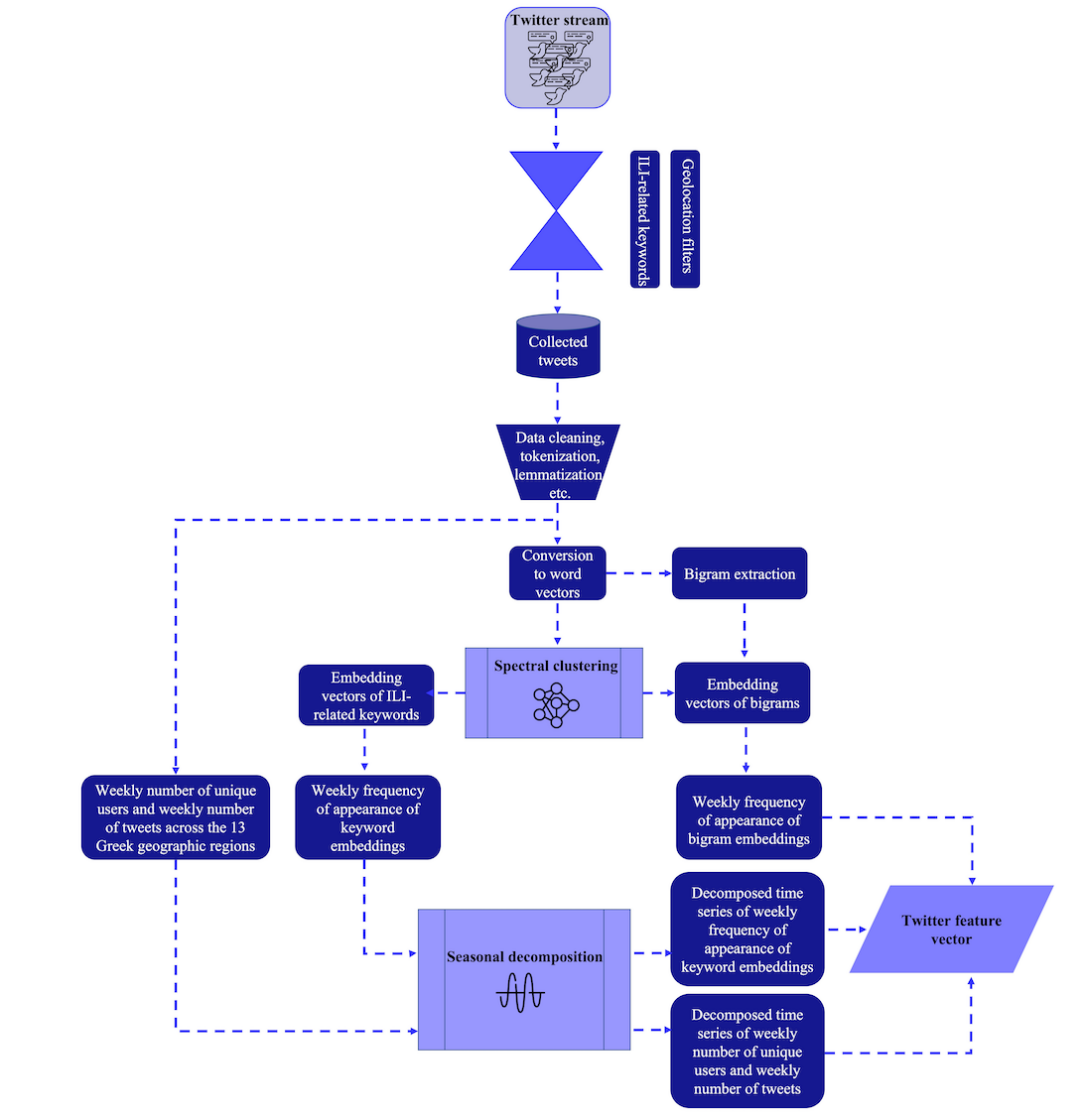
Flowchart of the implemented procedure to extract informative features from the collected Twitter data set. ILI: influenza-like illness.

Within the framework of this study, STD was applied to the weekly cluster-based frequencies of appearance of the ILI-related keyword embeddings as well as to the weekly number of unique Twitter users and the weekly number of tweets. The data collection was based on a weekly sampling frequency for each year of the decade of 2010 to 2019, which corresponded to 52 weeks. Considering the high dimension (ie, 1×124) of the weekly features corresponding to the cluster-based frequencies of appearance of bigram embeddings, these were excluded from the seasonal decomposition procedure to avoid a further increase in the dimension of the models’ input space. The adopted approach for the extraction of informative features from the obtained Twitter data set is illustrated in Figure 4.

#### Investigation of Feature Significance

A statistical analysis based on Pearson correlation and multivariate linear regression was performed to investigate the relationship between each of the considered factors and the number of ILI cases, as well as their effect on the progression of ILI cases. In particular, the number of hospital visits, the meteorological parameters used, and the frequency of appearance of the selected ILI-related keywords on a weekly basis throughout the decade of 2010 to 2019 were considered in the analysis. The obtained results, summarized in Table 1, revealed a statistically significant positive correlation between the weekly number of hospital visits and the weekly number of ILI cases (PCC >0.5; *P*<.05). Statistically significant correlations were also identified in the case of meteorological parameters across the 13 Greek regions, with the exception of precipitation in Central Greece and Central and West Macedonia, surface pressure in West Macedonia, and wind speed in Central Greece, as well as in the case of the frequency of appearance of all ILI-related keywords except for *otitis*, *asthma*, *diarrhea*, *dizziness*, *migraine*, *nausea*, *painkiller*, *paracetamol*, *sneeze*, *stomachache*, *symptoms*, and *shiver*. The multivariate linear regression analysis highlighted the statistically significant effect (*P*<.05) of the number of hospital visits and meteorological parameters on the number of weekly ILI cases. The regression analysis yielded similar results for the frequency of appearance of all ILI-related keywords—except for *asthma*, *migraine*, *paracetamol*, *sneeze*, and *stomachache*—which proved to be statistically significant in estimating the number of ILI cases.

**Table 1:** Investigation of feature significance through statistical analysis.

Data category	Pearson correlation	Multivariate linear regression
Hospital visits	*P*<.05	*P*<.05
Meteorological parameters	*P*<.05 for all meteorological parameters across the 13 geographical regions except for (1) precipitation in Central Greece and Central and West Macedonia, (2) surface pressure in West Macedonia, and (3) wind speed in Central Greece	*P*<.05 for all meteorological parameters across the 13 geographical regions
Frequency of appearance of ILI^a^-related keywords	*P*<.05 for all ILI-related keywords except for “otitis,” “asthma,” “diarrhea,” “dizziness,” “migraine,” “nausea,” “painkiller,” “paracetamol,” “sneeze,” “stomachache,” “symptoms,” and “shiver”	*P*<.05 for all ILI-related keywords except for “asthma,” “migraine,” “paracetamol,” “sneeze,” and “stomachache”

^a^ILI: influenza-like illness.

### Conceptual Framework

#### Overview

**Figure 5:**
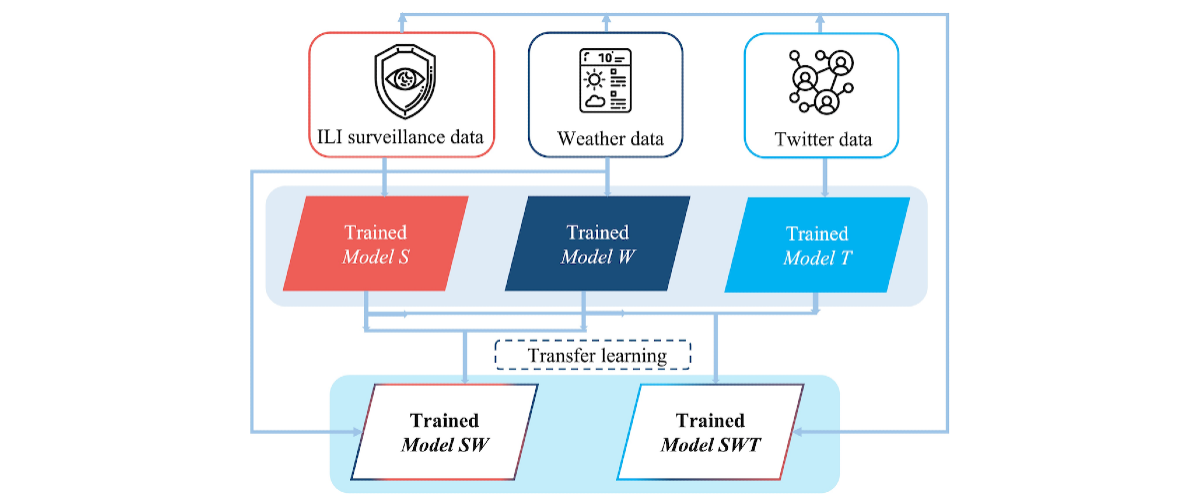
Conceptual framework of the proposed approach. Different models were developed by leveraging the obtained multisource data. ILI: influenza-like illness.

To efficiently handle the sequential and nonlinear nature of the multitude of collected data, LSTMs were leveraged owing to their inherent ability to capture long-term dependencies and identify complex patterns in time-series data. Along these lines, 3 LSTM-based primary models were developed, each receiving as input a specific data category (ie, ILI surveillance data, Twitter data, and weather data). The primary models were subsequently combined after the deployment of appropriate transfer learning (TL) strategies to develop combinatorial architectures and configurations of the trained primary models (TL-based combination models). The first model (*Model SW*) was based on the combination of the 2 primary models that considered the surveillance and weather data for composing their input space, whereas the second model (*Model SWT*) was developed by harnessing the combinatorial power of all the considered data sources through the use of the 3 primary models. A window size of 4 weeks was applied so that all models were fed with data from the previous 4 weeks. Figure 5 summarizes the different models that were developed to forecast the weekly number of ILI cases. The models are described in detail in the following sections.

#### Architectures of the Primary Models

ILI surveillance data, Twitter data, and weather data were first deployed separately to develop 3 primary models able to nowcast (estimation for the current week) and forecast (estimation for 1 and 2 weeks ahead) the weekly number of ILI cases in Greece based on the use of LSTMs. The first primary model (*Model S*) received as input the collected ILI surveillance data (ie, the weekly number of ILI cases and ILI-related hospital visits, which were represented by a 1×2 input vector). The second primary model (*Model W*) received as input the measurements of 8 weekly meteorological parameters for each of the 13 Greek geographical regions, corresponding to a 1×104 input vector. The third primary model (*Model T*) received as input the weekly features extracted from the Twitter data, that is, the weekly frequencies of appearance of bigram embeddings as well as the *seasonality*, *trend*, and *residual* components of (1) the weekly frequencies of appearance of the ILI-related keyword embeddings, (2) the weekly number of unique Twitter users for each of the 13 Greek geographical regions, and (3) the weekly number of tweets for each of the 13 Greek geographical regions, which composed a 1×224 input vector.

**Figure 6:**
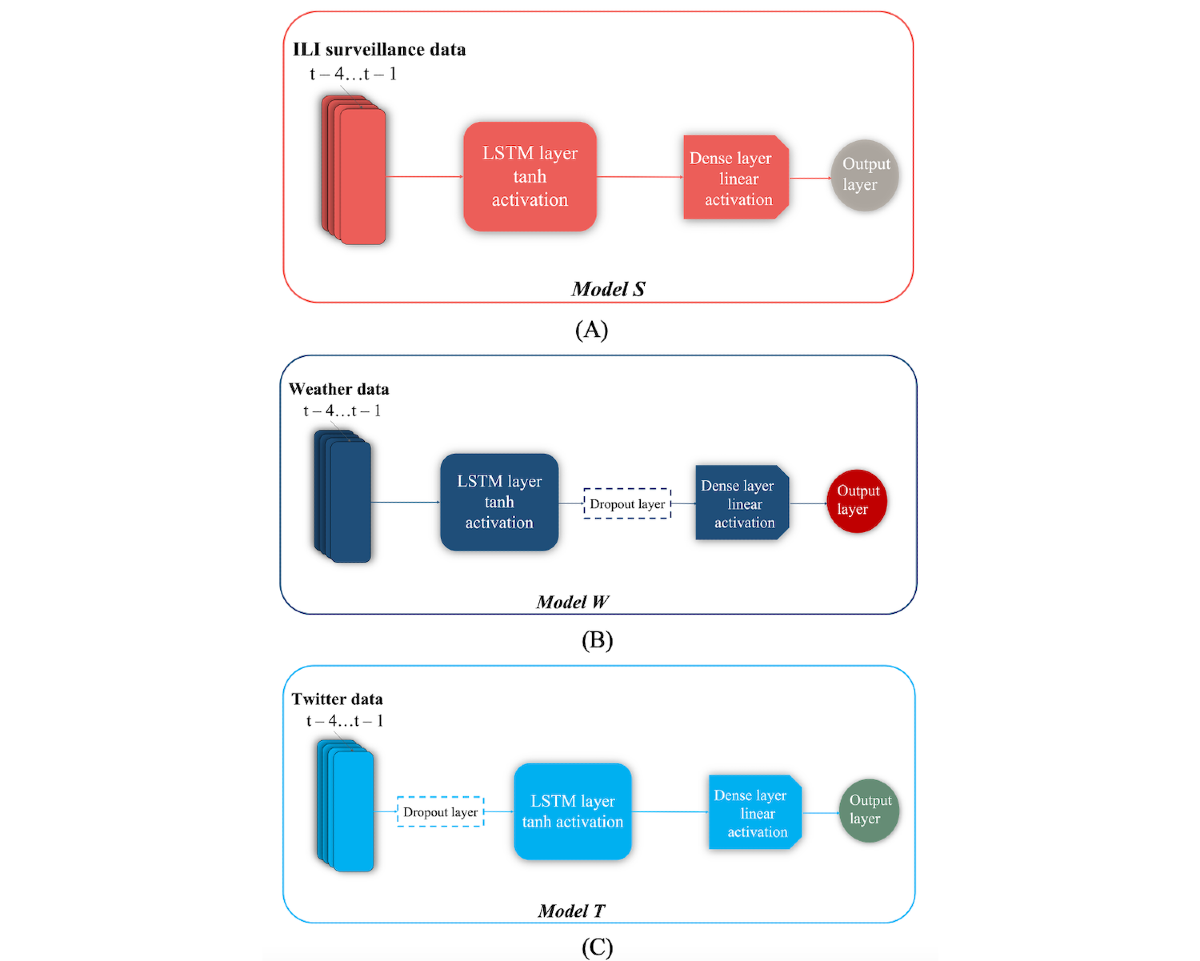
Architectures of the long short-term memory (LSTM)–based primary models. ILI: influenza-like illness.

To effectively handle the different categories of extracted features, the model architectures used for each primary model featured an LSTM layer that was followed by a fully connected output layer (dense layer) with a single node and a linear activation function. A dropout layer was applied during training to prevent overfitting. The position of the dropout layer was differentiated among the primary models based on the type and complexity of the considered features [51]. More specifically, for *Model W*, dropout was applied to the output values of the LSTM layer so as not to affect the data representations considered by the LSTM cells during weight training, whereas for *Model T*, dropout was applied to the input values of the LSTM layer to facilitate the memorization process given the large number of input instances and features. No dropout layer was used in the case of *Model S*. The architectures of the primary models are shown in Figure 6.

#### TL-Based Combination Model Architectures

The promising performance of epidemiological models that harness the power of alternative data sources along with the existing delays in the availability of traditional surveillance data reports, which usually hamper the real-time prognosis of ILI spread, motivated the exploration of combinations of traditional and alternative data sources for the development of reliable models able to accurately estimate the evolution of ILI cases. To this end, different TL approaches were adopted to leverage and combine the knowledge of the LSTM-based primary models to achieve enhanced predictive power. In total, 2 TL-based combination models were developed, each using a combinatorial architecture of the *Primary Models S*, *W*, and *T* (Textbox 2).

**Description of the 2 transfer learning (TL)–based combination models developed.**

**Figure 7:**
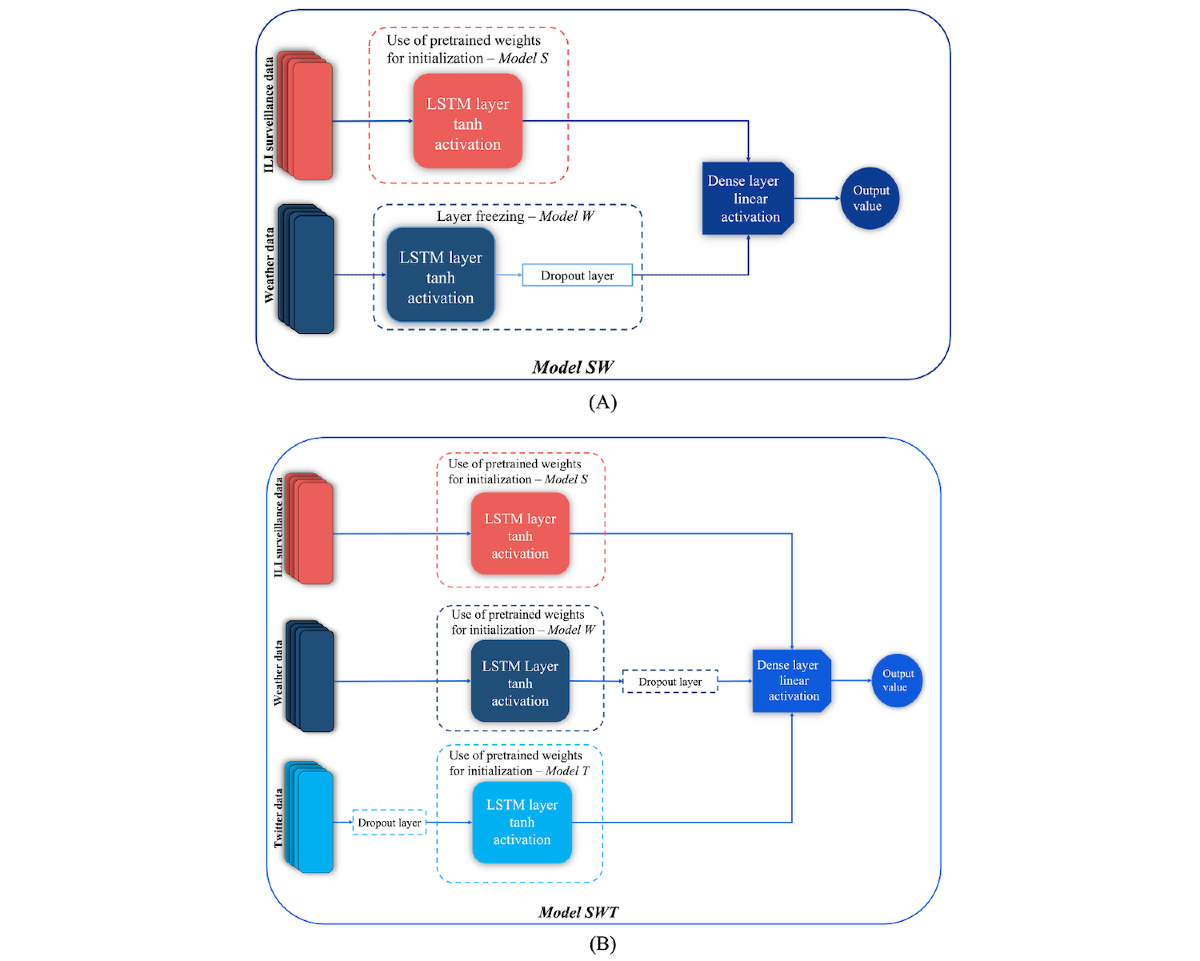
Architectures of the transfer learning–based combination models. ILI: influenza-like illness; LSTM: long short-term memory.

- *Model SW*
  The model was built upon the combination of *Primary Models W* and *S*. In particular, it comprised the long short-term memory (LSTM) layers of the 2 primary models, which were connected to a common, fully connected output layer with a single node and a linear activation function, as shown in Figure 7A. Each LSTM layer received as input the extracted features of the corresponding primary model’s data source. The high predictive performance of *Model W* combined with the observed time lag in the predictions produced by *Model S* in the validation set motivated the adoption of a hybrid TL approach during training using a layer freezing scheme and a weight initialization scheme. In particular, the weights of the pretrained LSTM layer of *Model W* were preserved, whereas the pretrained weights of the *Model S* LSTM layer were used for the initialization of the corresponding LSTM layer. Thus, *Model SW* was trained to update the weights of the LSTM layer of *Model S* and the weights of the new fully connected layer. During training, the hyperparameters of the LSTM layers of *Model W* and *Model S* were maintained.
- *Μodel SWT*
  The TL-based combination model (*Model SWT*) capitalized on the combination of the LSTM layers of *Models T, W,* and *S*. In particular, the LSTM layers of the 3 primary models were connected to a common, fully connected output layer with a single node and a linear activation function (Figure 7B). The extracted features of each data source were fed into the LSTM layer of the corresponding primary model. During training, the weights of the LSTM layers of the 3 primary models were initialized using the pretrained weights, which provided an optimal starting point for the training procedure. Thus, *Model SWT* was trained to update the weights of the 3 LSTM layers as well as those of the new fully connected layer. During training, the hyperparameters of the LSTM layers of the 3 primary models were preserved.

### Model Development

#### LSTM Units

LSTM is a variant of RNNs initially designed for addressing the problem of vanishing gradients, which in the case of RNNs hampers the learning of long data sequences. By incorporating a gating mechanism that controls the memorization process, LSTMs are able to efficiently handle sequential data, as demonstrated by their promising results in a wide range of classification and regression tasks [52]. In addition to the *hidden state* that exists in RNNs and constitutes a representation of the most recent time-step data, LSTMs introduce the *cell state*, which encodes knowledge aggregated from all previous time steps, thus yielding long-term memory capabilities in the network. The structure of the LSTM cell comprises 3 gates (ie, the forget gate, the input gate, and the output gate) that regulate the information flow in the LSTM cell [53]. The cell state of the current time step *C*_t_ is calculated based on the equation 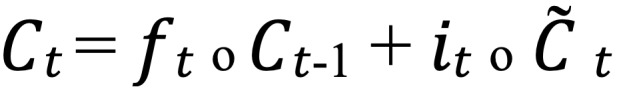 (5), where *C_t–1_* is the cell state of the previous time step; *f_t_* and *i_t_* represent the forget and input gates, respectively; and *C* ~_*t*_ is a candidate vector, which includes a set of new values that could be used to update the cell state. Information from the previous hidden state and the current input are fed into the forget gate and the input gate, which determine the information that is neglected by or added to the cell state, respectively, according to the following equations:


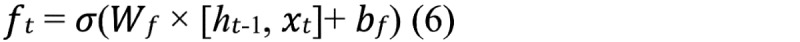



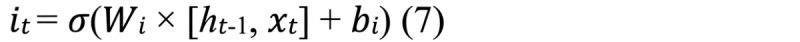


In Equations 6 and 7, *x*_*t*_ and *h*_*t*–_*_1_* represent the current input vector and the hidden vector of the previous state, respectively. The candidate vector is calculated as:


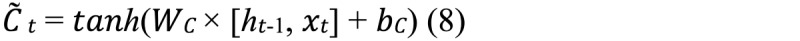


The output gate *o* regulates the information that will be passed on to the hidden state and updates the hidden state from *h_t–1_* to *h_t_* according to the following equations:


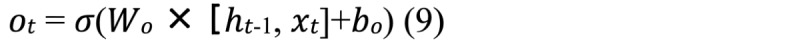



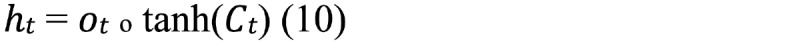


In Equations 5 to 10, σ is the sigmoid activation function, and *W_x_* and *b_x_* (*x*=*f, i, C*) are weight matrices and bias vector parameters, respectively.

In this study, the development of the LSTM-based primary models and TL-based combination models was based on the use of multivariate architectures, receiving multiple features as input to estimate the number of ILI cases.

#### Hyperparameter Tuning

A brute-force grid-search approach with parallelized performance evaluation was applied for the optimal tuning of the hyperparameters of the LSTM-based primary models. Textbox 3 summarizes the set of LSTM hyperparameters that were selected to be investigated. The combinations of hyperparameters that achieved minimization of the mean square error (MSE) in the validation set were first identified, and the subset of hyperparameter combinations leading to the derivation of predictions that were significantly correlated (*P*<.05) to the actual ILI cases in the validation set were eventually considered for tuning the LSTM-based primary models. An early stopping criterion was also applied to avoid overfitting based on the interruption of the training phase in case error reduction was not observed for 20 consequent epochs. The applied hyperparameters for each primary model were as follows: (1) *Model S* featured an LSTM layer with 8 nodes (no dropout was applied during training); (2) *Model W* featured an LSTM layer with 60 nodes, and a dropout layer with a factor equal to 0.1 was applied during training; and (3) *Model T* featured an LSTM layer with 90 nodes, and a dropout layer with a factor equal to 0.1 was applied during training.

**Variation of the hyperparameters of the long short-term memory (LSTM)–based primary models.**

- Hyperparameter and value range
  Optimizer: Adam, Nadam
  LSTM window size (in weeks): 2, 3, 4
  Batch size: 1, 4, 8, 16
  Dropout: 0, 0.4 with step=0.1
  Epochs: 30, 80 with step=10
  Weight initialization: Glorot normal, Glorot uniform
  Activation function: sigmoid, tanh
  Number of nodes: 2, 4, 8, 10, 15, 20, 25 and 30 to 100 with step=10

The number of training epochs was eventually set to 50, and the batch size was selected to be 1 across all models. A window size of 4 weeks was adopted. All input features were normalized to the range of −1 to 1.

### Evaluation Framework

The generalization abilities of the developed models were evaluated by applying 10-fold cross-validation. The division of the collected data into 10 sets of identical size was performed according to their reference year. The data samples for each year of the period under study comprised the set of a corresponding fold. In this regard, in each iteration of the 10-fold cross-validation, the models’ performance was evaluated on data from a year to which the models were not exposed during the training phase. This process enabled model evaluation at a yearly level, thus leading to the establishment of a more reliable evaluation framework. During the training stage of each iteration of the cross-validation, the training set was partitioned into training and validation subsets based on a 90/10 ratio to facilitate hyperparameter selection. The predictive performance of the LSTM-based primary models and TL-based combination models was evaluated for different forecasting horizons (FHs; ie, current week, 1 week ahead, and 2 weeks ahead) using the PCC, mean absolute error (MAE), MSE, and root MSE (RMSE), which are traditionally deployed for performance assessment in terms of accuracy and robustness [54].

## Results

### Overview

The nowcasting (estimation for the current week [FH=0]) and forecasting (estimation for 1 week [FH=1] and 2 weeks [FH=2] ahead) performances of the LSTM-based primary models and TL-based combination models were evaluated based on the mathematical criteria described in the previous section. Table 2 presents the average and SD values of MAE, MSE, RMSE, and PCC that were calculated by considering the 3 FHs within the framework of the 10-fold cross-validation. To compare the averaged evaluation criteria, the pairwise 2-tailed *t* test was used, and the cutoff .05 was considered as the level of significance.

**Table 2:** Evaluation of the models’ forecasting performance for different forecasting horizons (FHs). Criteria values were calculated by averaging the values corresponding to the testing sets generated by the 10-fold cross-validation.

FH and model		MAE^a^, mean (SD)		MSE^b^, mean (SD)	RMSE^c^, mean (SD)		PCC^d^, mean (SD)			
**Current week (FH=0)**										
	Model S	0.090 (0.020)	0.0178 (0.009)			0.125 (0.028)		0.896 (0.060)		
	Model W	0.070 (0.020)	0.012 (0.005)			0.103 (0.024)		0.922 (0.031)		
	Model T	0.206 (0.072)	0.082 (0.041)			0.259 (0.076)		0.689 (0.183)		
	Model SW	0.073 (0.018)	0.012 (0.006)			0.102 (0.027)		0.923 (0.041)		
	Model SWT	*0.069 (0.012)* ^e^	*0.010 (0.003)*			*0.097 (0.019)*		*0.923 (0.037) *		
**1 week ahead (FH=1)**										
	Model S	0.115 (0.027)	0.0291 (0.014)			0.160 (0.037)		0.849 (0.067)		
	Model W	0.090 (0.020)	0.024 (0.011)			0.145 (0.034)		0.865 (0.058)		
	Model T	0.204 (0.080)	0.090 (0.050)			0.264 (0.086)		0.667 (0.164)		
	Model SW	0.0927 (0.020)	0.023 (0.010)			0.141 (0.033)		*0.887 (0.029) *		
	Model SWT	*0.0833 (0.017)*	*0.019 (0.008)*			*0.127 (0.030)*		0.878 (0.059)		
**2 weeks ahead (FH=2)**										
	Model S	0.121 (0.023)	0.035 (0.027)			0.159 (0.037)		0.794 (0.065)		
	Model W	0.122 (0.029)	0.048 (0.036)			0.144 (0.034)		0.801 (0.058)		
	Model T	0.286 (0.138)	0.173 (0.161)			0.264 (0.086)		0.604 (0.155)		
	Model SW	0.121 (0.025)	0.039 (0.019)			0.140 (0.034)		0.795 (0.063)		
	Model SWT	*0.104 (0.014) *	*0.028 (0.011)*			*0.128 (0.029)*		*0.822 (0.067)*		

^a^MAE: mean absolute error.

^b^MSE: mean square error.

^c^RMSE: root mean square error.

^d^PCC: Pearson correlation coefficient.

^e^Italics indicate the performance of Model SWT.

### Performance of Primary Model and TL-Based Combination Model

It can be seen that the *Primary Models S* and *W* yielded a satisfactory performance, as reflected by the low MAE, MSE, and RMSE values and the high PCC values that were obtained across the 3 FHs. Statistically significant differences were observed between *Model T* and the rest of the primary models in terms of the applied evaluation criteria for all FHs, thus demonstrating the low predictive power of Twitter features in the collected data set. No other statistically significant differences in terms of performance were identified within the framework of the pairwise comparison.

The deployment of combinatorial architectures, which were based on different combinations of the LSTM-based primary models through TL, contributed to increasing the achieved forecasting performance. More specifically, the consolidation of weather and surveillance data through the combination of the architectures of the *Primary Models S* and *W* (*Model SW*) demonstrated equivalent performance to *Model W* but led to better performance with respect to *Model S* in terms of the absolute average values of most of the evaluation criteria. This observation motivated the investigation of the potential of enriching the input space with data from Twitter, despite the low performance of the primary *Model T*, through the development of *Model SWT.* Although no statistically significant differences in terms of performance were observed, *Model SWT* achieved improved forecast accuracy compared with all the other models across the 3 FHs.

**Figure 8:**
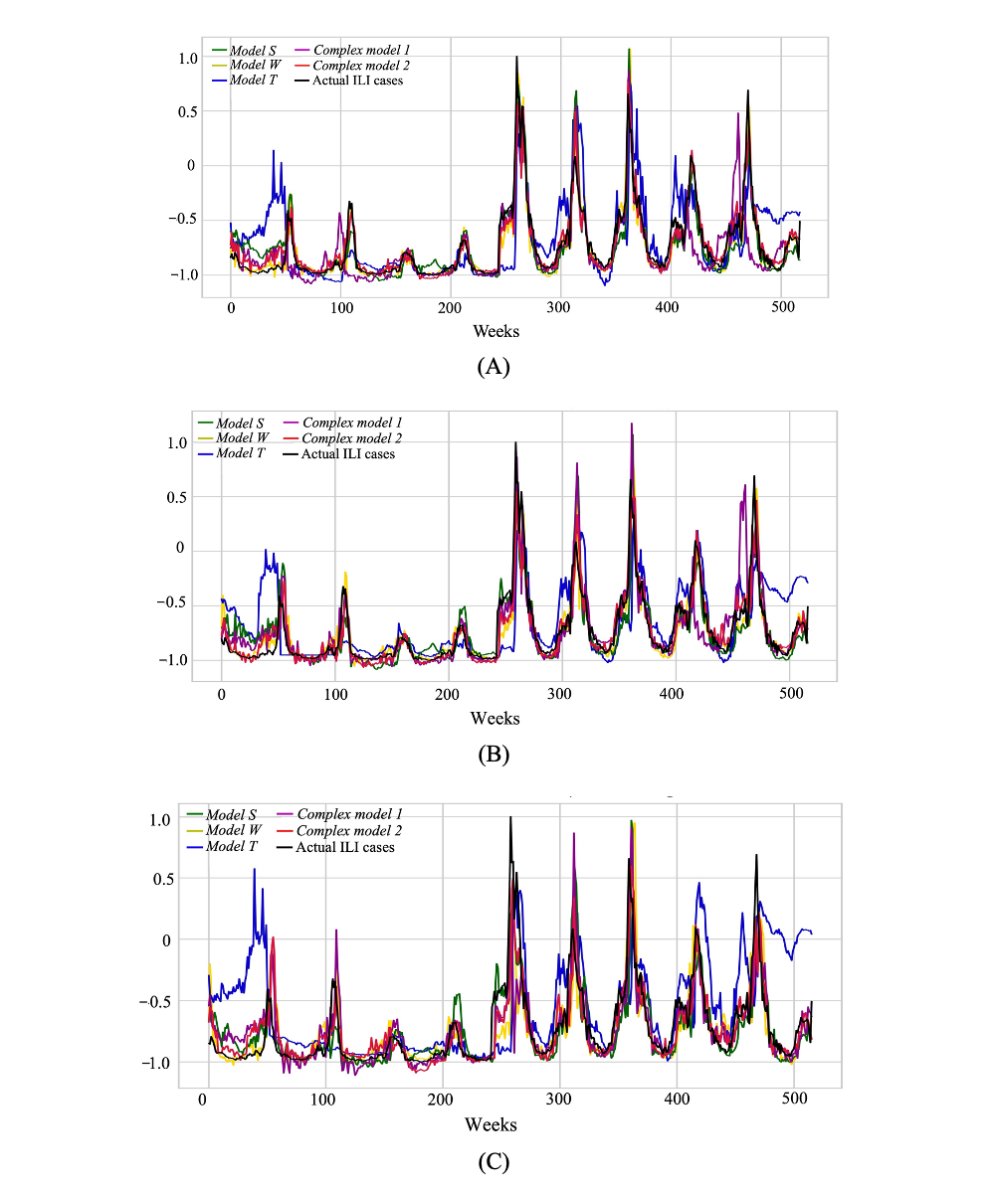
The obtained predictions of the developed primary and transfer learning–based combination models versus the actual influenza-like illness (ILI) cases for the 520 weeks of the decade of 2010 to 2019. (A) nowcasting, (B) forecasting—1 week, and (C) forecasting—2 weeks.

**Figure 9:**
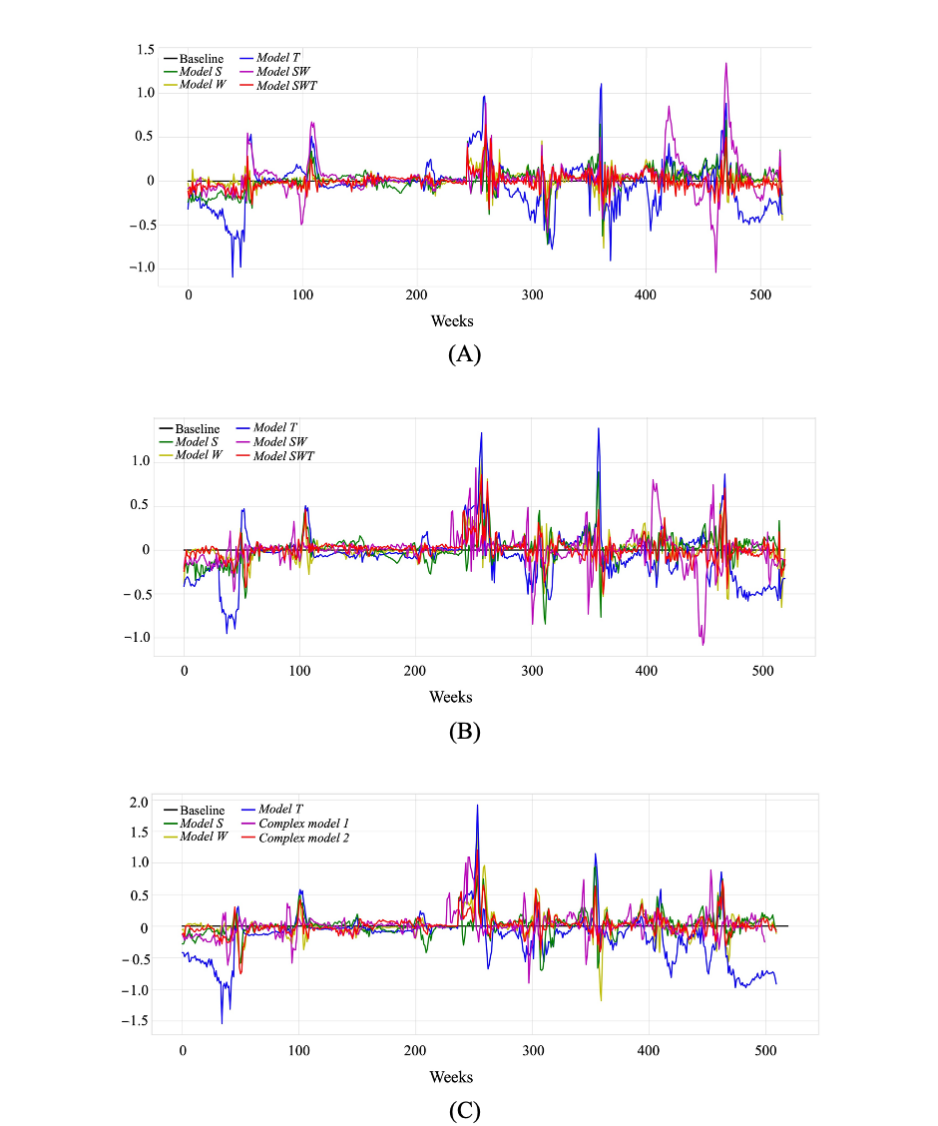
The prediction error of the developed primary and transfer learning–based combination models, defined as the difference between the predicted and actual weekly influenza-like illness cases, that was calculated for the 520 weeks of the decade of 2010 to 2019. (A) nowcasting, (B) forecasting—1 week, and (C) forecasting—2 weeks.

**Figure 10:**
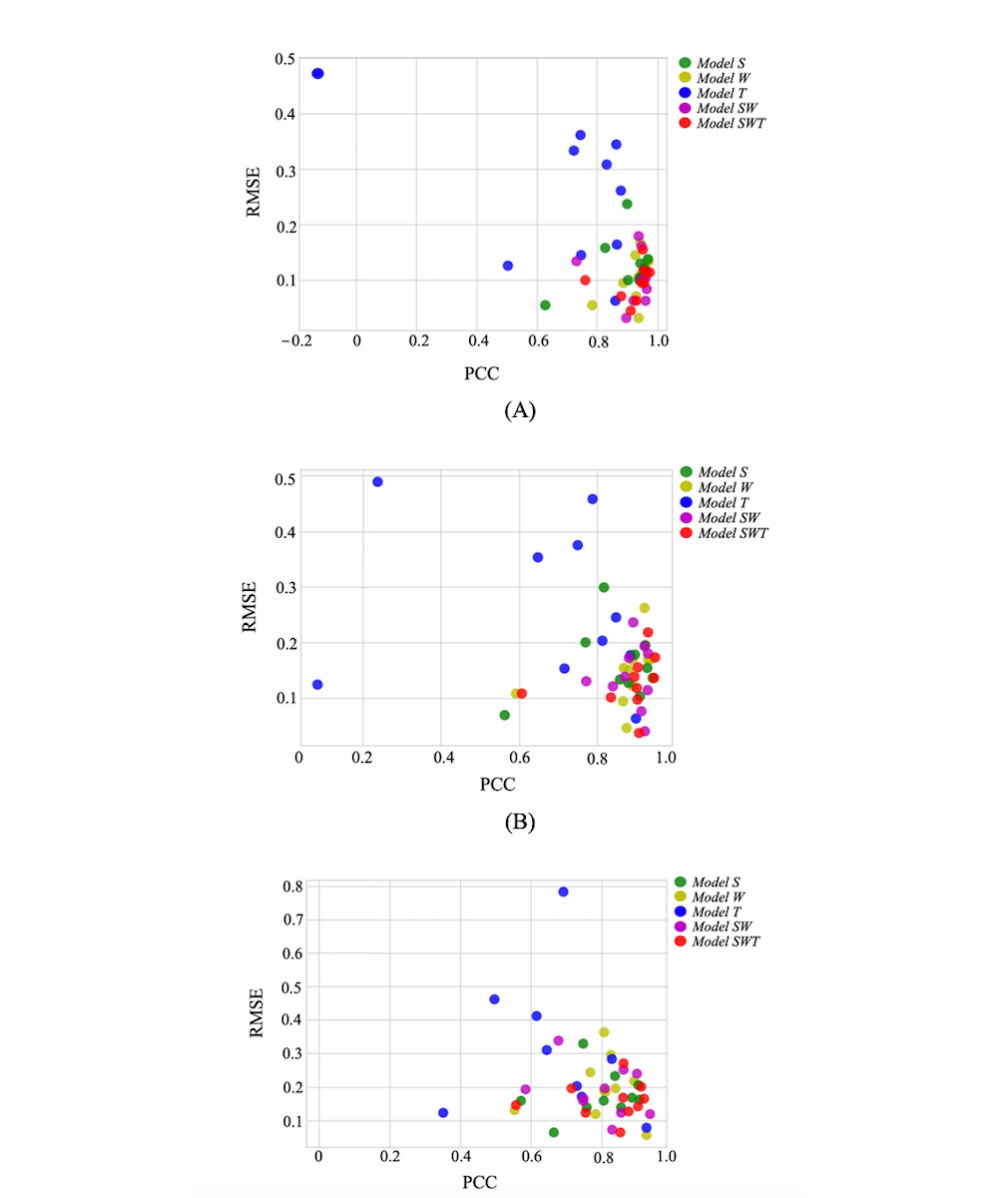
Performance of the developed primary and transfer learning–based combination models in terms of the root mean square error (RMSE) and the Pearson correlation coefficient (PCC) that were calculated for the decade of 2010 to 2019. Each dot represents the obtained results in the corresponding fold (year). (A) nowcasting, (B) forecasting—1 week, and (C) forecasting—2 weeks.

The real-time (nowcasting) predictions of weekly ILI cases as well as the predictions 1 and 2 weeks in advance, which were produced by the primary and TL-based combination models, are presented with respect to the actual weekly ILI cases in Figure 8. The corresponding prediction errors, defined as the difference between the predicted and actual weekly ILI cases, which were calculated for the 3 FHs throughout all weeks in the decade under study, are shown in Figure 9. It is observed that, although the performance of the primary and combination models varied across the weeks, *Model SWT* exhibited steadily lower prediction errors and reduced time lag between the predicted and true values across all FHs when compared with the other models. This is also illustrated in Figure 10, which contrasts the PCC and RMSE of the primary and combination models obtained for the 3 FHs in each repetition of the 10-fold cross-validation scenario. It is shown that the combination models outperformed the primary models in most of the data splits. *Model SWT* yielded the highest forecast accuracy across all folds, resulting in high PCC values and low RMSE values, whereas *Model T* demonstrated the worst performance among all models.

## Discussion

### Principal Findings

Although the existing epidemiological models of ILI spread are mainly based on the use of ILI surveillance data and mathematical modeling, the potential of harnessing alternative data sources along with machine learning techniques has been explored in a variety of studies. In this work, the combined use of data from Twitter, meteorological databases, and surveillance system reports along with LSTMs was investigated for the first time, and different prediction models of ILI spread were developed and comparatively assessed. The models’ input space was composed of a variety of features derived from ILI historical data, meteorological parameters, and ILI-related tweets. In the case of tweets, a novel preprocessing approach was adopted with the aim of extracting highly informative features to enhance model learning. Simple and more sophisticated LSTM-based architectures were deployed to efficiently handle the various data combinations. Considering the existence of a 1-week lag between the publication of ILI surveillance reports and actual ILI activity, a window size of 4 weeks was applied in this study, and all models were fed with data from the previous 4 weeks. Thus, the obtained predictions were made available as soon as the data of the previous weeks were captured, providing estimates of ILI spread faster than current practice allows. Greece was selected as a use case for the proposed approach, and a strict and reliable evaluation framework enabling the assessment of the models’ generalization capabilities on a yearly basis was applied.

It was demonstrated that the prediction model that learned from meteorological parameters (*Model W*) yielded a comparable performance with that of the model that was trained with ILI historical data (*Model S*). In contrast, the predictive power of Twitter features (*Model T*) proved to be lower with respect to ILI historical data and weather data. The low performance of *Model T* was mainly attributed to the limited, nonuniform, and ever-decreasing use of Twitter across the geographic regions of Greece, which introduced challenges in the collection of representative sets of tweets. However, it was observed that *Model T* presented an increased sensitivity in capturing changes in the rate of progression of ILI spread with respect to *Model S* and *Model W*, as illustrated in Figure 8. This remark motivated the development of combination models by leveraging combinations of the primary models’ architectures based on the application of TL approaches.

The deployment and comparison of the multisource, heterogeneous data combinations highlighted the increased effectiveness of TL-based combination models against simpler ones, which received as input the features that were extracted from a single data category. In particular, *Model SWT* achieved superior performance over the rest of the models and exhibited high forecast accuracy, reflecting its ability to provide reliable estimations of ILI spread on a weekly basis. The consolidation of Twitter data in *Model SWT* contributed to performance improvement with respect to *Model SW*, thus revealing the ability of the combination model to leverage the Twitter extracted features more efficiently than *Model T*. It was shown that models based on the combined use of Twitter data, weather data, and ILI surveillance data outperformed models that relied solely on ILI surveillance data, thus indicating the potential of these alternative public sources to enhance accurate and reliable prediction of ILI spread.

Overall, the proposed approach provides a framework for integrating traditional parameters considered in epidemiology with large-scale social patterns revealed through social media data toward the development of models for monitoring and forecasting ILI epidemics. To overcome data and platform variations and facilitate the generalizability of this approach, this study explored the use of ILI surveillance data, weather data, and Twitter data, which can be used separately or in combination across different use cases given their broad availability. It was shown for the first time that harnessing the combinatorial power of these multisource data along with advanced machine learning techniques can increase the accuracy of prediction models of ILI spread. Although the validation of these models was based on the use of data from Greece, the satisfactory performance of *Model SWT* highlighted its ability to identify trends and confirm observations from traditional surveillance approaches, thus providing evidence of the potential of the proposed approach to be used within the framework of different contexts in terms of populations, locations, and infectious diseases.

In view of the limited number of studies focusing on the analysis of data sets containing Greek tweets, the optimal use of the collected Twitter data for the prediction of ILI spread presented substantial interest while also introducing a considerable challenge. The character limit imposed by Twitter in combination with the circumlocutory nature and semantic complexity of the Greek language may limit the level of informative content included in Twitter posts. To address this challenge, emphasis was placed on the careful selection of ILI-related keywords, facilitating the collection of representative data, as well as on the investigation of different preprocessing techniques for the extraction of highly informative features. Although frequently used text features such as word n-grams and term frequency-inverse document frequency scores were first explored, their use resulted in low predictive performance, thus underlining the need to apply more sophisticated preprocessing methods.

Indeed, the adoption of a tailored method based on spectral clustering proved beneficial in addressing the complex nature of the Greek language and the difficulties introduced because of the limited Twitter use and significantly enhanced the models’ knowledge space, as indicated by the performance of *Model SWT*. Along these lines, despite this study’s focus on Twitter and the Greek language, this technique could be generalized to other use cases as well, comprising combinations of social media platforms that feature text posts characterized by similar limitations in the collected corpus and languages with increased linguistic complexity, which cannot be effectively handled by standard NLP techniques. Subsequently, the proposed approach for the development of prediction models of ILI spread could be extended to other locations, populations, and social media platforms to advance the existing disease surveillance practice for ILI and other infectious diseases, including COVID-19. Standard or more complex preprocessing techniques could be applied for extracting text features depending on the social media platform considered and the language of the use case.

### Comparison With the State of the Art

A fair comparison between the obtained results and those reported in the literature was not feasible as the use of Twitter data in combination with weather data and ILI surveillance data has not been previously explored. Various studies focusing on the development of ILI prediction models based on the use of weather data or social media data, including Twitter data, have highlighted the high predictive power of these data sources with respect to ILI surveillance data, as indicated by the reported values for RMSE and PCC, which ranged from 0.54 to 0.98 [26,29,55]. By (1) formulating a richer feature space to account for decreasing and increasing trends in ILI spread, (2) introducing a novel preprocessing strategy to extract informative features of Twitter data, and (3) deploying efficient TL-based techniques to capitalize on the knowledge of simpler models, the proposed approach managed to achieve equivalent or superior performance to that reported in the literature, even in the case of longer FHs. It is also noteworthy that, in most studies, the validation method was based on split sampling, which in turn might have introduced bias in the analysis. In this work, the applied evaluation framework provided a reliable measure of the models’ generalization capabilities as each fold in the 10-fold cross-validation method corresponded to a specific year, thus being independent from the other folds.

### Limitations

Potential limitations of this study are mainly associated with the selection of Greece as a use case for model development and evaluation. Although the focus on a single country could facilitate the generation of representative data on the patterns of ILI spread at a national level, the decreasing and nonuniform use of Twitter across Greece led to the collection of a limited tweet data set that could not accurately reflect the number of ILI cases. To investigate the influence of variations in Twitter use on the models’ performance throughout the decade under study, potential correlations were explored through Pearson correlation analysis between Twitter use, as reflected in the number of collected tweets and the percentage of Twitter users in Greece [56], and the performance of *Model SWT* in terms of the RMSE and PCC. The obtained results did not reveal statistically significant correlations between RMSE and Twitter use, but a statistically significant positive correlation (*P*<.05) was observed between Twitter use and PCC in the case of the 1-week FH, thus suggesting that fluctuations in the number of Twitter users may have affected the accuracy level of the produced predictions. The ability of Twitter data to enhance predictive performance despite the previous finding provided evidence regarding the crucial role of the quality of the collected corpus and the effectiveness of the applied feature extraction methods in untapping the potential of Twitter data.

Another important aspect is the generalizability of Twitter data so that they accurately reflect incidents in the general population. In particular, although the demographic characteristics of Twitter users, especially those that would post about health-related issues, are unknown and difficult to estimate, according to the available reports on Twitter demographics, most of the Twitter audience falls within the age range of 25 to 34 years (38.5%) and lives in urban areas (27%) [57]. Considering that ILI epidemics depend on the immunity of the general population and that high-risk groups mainly comprise children, older adults, and people with chronic conditions, it becomes evident that Twitter users cannot reflect the entire spectrum of patients with ILI. In particular, ILI infections in older individuals, children, and people living in rural areas may not be adequately represented in the Twitter data set except for tweets referring to infections of individuals from the users’ family or social circle. The aforementioned limitations may have undermined the positive impact of the consideration of Twitter data on the performance of *Model SWT*, thus not unveiling the full potential of this approach. However, the satisfactory performance of *Model SWT* on a use case characterized by the aforementioned limitations indicated the ability of the approach to meet the requirements of other use cases, referring to different locations and populations, and capture the ILI epidemic spread independently of the corresponding levels of Twitter use.

### Future Directions

Future work concerns the validation of the proposed methods on larger data sets (eg, by considering data from other countries) with the ultimate goal of providing a well-validated prediction model able to aid ILI epidemiological surveillance by harnessing the real-time availability of social media and weather data. In this direction, the consideration of different countries with more intense Twitter use will be investigated to improve the models’ performance. Focus will also be placed on exploring the potential of *Model SWT* to facilitate monitoring of the progression of COVID-19 spread. Considering the availability of a multitude of COVID-19–related data sources and the plurality of evidence regarding the effectiveness of using weather and social media data for the development of epidemiological models for COVID-19 surveillance, the proposed approach will be appropriately adjusted with the aim of providing accurate predictions of COVID-19 cases [21,58,59]. To enhance users’ trust and facilitate the adoption of these models in disease surveillance practice, state-of-the-art explainability techniques need to be explored for the provision of explanations of the models’ decisions, with the ultimate goal of delivering a retraceable, understandable, and human interpretable approach enabling informed disease surveillance.

### Conclusions

This study highlights the potential of leveraging alternative data sources for the development of sophisticated models able to enhance accurate and reliable prediction of ILI spread. It was demonstrated that the combined use of weather and Twitter data can provide a significant and cost-effective supplement to traditional disease surveillance systems even in the case of regions characterized by lower Twitter use. Although the development and validation of these models were based on the use of data from Greece, the obtained results indicate the potential of the proposed approach to be generalized to other locations, populations, and social media platforms to support the surveillance of infectious diseases.

Considering the important role of digital solutions complementing traditional public health approaches in the era of the COVID-19 pandemic, the proposed approach can serve as a means of routine surveillance during nonemergency times as well as aid the implementation of rapid response strategies during disease outbreaks. By enabling timely identification of patterns and accurate forecasting of the evolution of disease spread, this work may contribute to advancing the field of infodemiology and facilitate the creation of digital surveillance solutions at a national and international level with the ultimate goal of improving capacity and preparedness for future epidemics.

## Abbreviations

API: application programming interface
DDD: digital disease detection
FH: forecasting horizon
ILI: influenza-like illness
LSTM: long short-term memory
MAE: mean absolute error
MSE: mean square error
NLP: natural language processing
PCC: Pearson correlation coefficient
RMSE: root mean square error
RNN: recurrent neural network
STD: seasonal trend decomposition
SVM: support vector machine
TL: transfer learning

## Data Availability

The data sets generated and analyzed during this study are available from the corresponding author upon reasonable request.
